# Regulation of the E/I-balance by the neural matrisome

**DOI:** 10.3389/fnmol.2023.1102334

**Published:** 2023-04-18

**Authors:** Cornelius Mueller-Buehl, David Wegrzyn, Juliane Bauch, Andreas Faissner

**Affiliations:** Department of Cell Morphology and Molecular Neurobiology, Ruhr-University Bochum, Bochum, Germany

**Keywords:** tenascin-c, perineuronal nets, parvalbumin, extracellular matrix, schizophrenia, excitation-inhibition (E/I) balance, lecticans, synapse

## Abstract

In the mammalian cortex a proper excitatory/inhibitory (E/I) balance is fundamental for cognitive functions. Especially γ-aminobutyric acid (GABA)-releasing interneurons regulate the activity of excitatory projection neurons which form the second main class of neurons in the cortex. During development, the maturation of fast-spiking parvalbumin-expressing interneurons goes along with the formation of net-like structures covering their soma and proximal dendrites. These so-called perineuronal nets (PNNs) represent a specialized form of the extracellular matrix (ECM, also designated as matrisome) that stabilize structural synapses but prevent the formation of new connections. Consequently, PNNs are highly involved in the regulation of the synaptic balance. Previous studies revealed that the formation of perineuronal nets is accompanied by an establishment of mature neuronal circuits and by a closure of critical windows of synaptic plasticity. Furthermore, it has been shown that PNNs differentially impinge the integrity of excitatory and inhibitory synapses. In various neurological and neuropsychiatric disorders alterations of PNNs were described and aroused more attention in the last years. The following review gives an update about the role of PNNs for the maturation of parvalbumin-expressing interneurons and summarizes recent findings about the impact of PNNs in different neurological and neuropsychiatric disorders like schizophrenia or epilepsy. A targeted manipulation of PNNs might provide an interesting new possibility to indirectly modulate the synaptic balance and the E/I ratio in pathological conditions.

## 1. Development of GABAergic interneurons

### 1.1. Local GABAergic circuit neurons contribute to a proper E/I-balance

In the mammalian cortex two distinct classes of neurons enable its remarkable functions and properties (Cadwell et al., 2019; Anastasiades and Carter, 2021). The first one is formed by layer-specific projection neurons which induce a depolarization at the postsynapse by a release of glutamate into the synaptic cleft (Lodato et al., 2011; Marin and Muller, 2014; Berg et al., 2021; Matho et al., 2021). On the other hand, local circuit neurons form the second class and make up a proportion of 20% in the cortex (Hendry et al., 1987). In contrast to the projection neurons, local circuit neurons release the neurotransmitter GABA into the surrounding tissue. At the postsynapse, GABA mainly acts on ionotropic GABA_A_-receptors inducing a hyperpolarization of the contacted neuron (Sallard et al., 2021). A proper functionality of these GABAergic interneurons is essential for the establishment and maturation of neuronal circuits during the development and furthermore for the maintenance of an E/I-balance in matured networks. Interestingly, recent findings demonstrate that an interaction between subtypes of projection neurons with interneurons ensures a proper integration of the latter ones in the developing cortex (Lodato et al., 2011; Wester et al., 2019). Other important aspects of GABAergic interneurons were already addressed in several previously published review articles (Markram et al., 2004; Le Magueresse and Monyer, 2013). Here, studies of the last decades revealed that GABAergic interneurons are *inter alia* necessary for the establishment of cortical circuits (Roux and Buzsaki, 2015; Anastasiades et al., 2016; Tuncdemir et al., 2016; Cardin, 2018) proper brain oscillations (Bartos et al., 2007; Keeley et al., 2017; Kalemaki et al., 2018; Antonoudiou et al., 2020) and firing patterns (Couey et al., 2013; Miao et al., 2017).

### 1.2. Origin and subtypes of cortical GABAergic interneurons

Identifying the origins of cortical GABAergic interneurons was the subject of several studies. Here, it was shown that the medial and the caudal part of the ganglionic eminence are the main sources of interneuron progenitors which tangentially migrate in the expanding cortex during the embryonic development (Xu et al., 2004; Wonders and Anderson, 2006; Miyoshi and Fishell, 2011; Lim et al., 2018; Yu et al., 2021). Furthermore, two distinct interneuron lineages could be identified *via* retroviral labelling techniques on human organotypic slice cultures (Letinic et al., 2002). The study revealed that 65% of cortical GABAergic interneurons express the transcription factors distal-less homeobox-1/2 (Dlx1/2) as well as mammalian achaete-scute homolog-1 (Mash-1) and originate from Mash-1-expressing progenitors localized in the ventricular and subventricular zone of the dorsal forebrain (Letinic et al., 2002). The remaining 35% of the cortical interneurons are positive for Dlx1/2 but not for Mash-1 indicating a second interneuron lineage. The importance of Dlx-family members for the cortical interneuron population was verified in different studies. Here, conditional knockouts of *Dlx1*, *Dlx2*, and *Dlx1/2* induced abnormalities on a dendritic and synaptic level affecting especially the parvalbumin-expressing subpopulation of interneurons (Pla et al., 2018). Furthermore, the *Dlx1/2* double knockout condition prevented the differentiation of neural progenitor cells in the dorsal lateral ganglionic eminence and resulted in a failure of olfactory bulb interneurons (Guo et al., 2019). Importantly it has been observed that Dlx2 drives the expression of Gad1, Gad2, and VGAT and both, Dlx1 and Dlx2, induce the synthesis of GABA (Pla et al., 2018). A complete loss of Dlx1 induced a subtype-specific loss of interneurons with accompanying epileptic seizures (Cobos et al., 2005). Deficits concerning Dlx1/2 also led to an impaired fear conditioning (Mao et al., 2009) and a disruption of progenitor migration (Le et al., 2007).

Similarly, the ganglionic eminence was identified as the main source of cortical GABAergic interneurons in rodents. Here, 50–60% of the cortical interneuron population are supposed to originate from the medial ganglionic eminence (Butt et al., 2005; Wonders et al., 2008; Kelsom and Lu, 2013) while another 30–40% originate from the caudal ganglionic eminence (Nery et al., 2002; Kelsom and Lu, 2013). The embryonic preoptic area, a part of the hypothalamus, contributes furthermore to approximately 10% of the cortical GABAergic interneurons (Gelman et al., 2009, 2011; Niquille et al., 2018; Symmank and Zimmer-Bensch, 2019). After the migration and positioning in the developing cortex the interneuron progenitors differentiate and mature into several interneuron subtypes. It is supposed that there are 20 interneuron subtypes in the cortex (Somogyi and Klausberger, 2005; Klausberger and Somogyi, 2008; Fishell and Rudy, 2011; Kelsom and Lu, 2013). The different types of interneurons are traditionally categorized according to the expression of subtype-specific markers and their morphology. Here, 40% of the cortical interneurons express the Ca^2+^-binding protein parvalbumin and can be differentiated into basket cells and chandelier cells. Further 30% of the cortical interneurons are positive for the neuropeptide somatostatin and include martinotti and non-martinotti cells (Rudy et al., 2011; Kelsom and Lu, 2013; Tremblay et al., 2016). Martinotti cells and non-martinotti cells in the brain demonstrate distinct connectivity patterns and unique molecular profiles. In the rat somatosensory cortex, all martinotti cells express somatostatin, but not parvalbumin or vasoactive intestinal peptide. Some of them also expressed Calbindin, calretinin or neuropeptide Y (Wang et al., 2004). Cell bodies of these interneurons’ resident in cortical layer II/III and layer V and axonal arbors of these interneurons are described to extend to layer I (Karube et al., 2004; Xu et al., 2013). The combination of whole-cell recordings from pyramidal cells and interneurons, along with morphological reconstructions, has led to the identification of martinotti cells as the mediating interneurons in this feedback pathway. The pathway may play a central role in regulating cortical activity (Silberberg and Markram, 2007). In contrast, non-martinotti cell bodies in the barrel cortex reside in layer IV, Vb, and Vb/VI (Munoz et al., 2017). They mainly innervate parvalbumin interneurons in layer IV (Xu et al., 2013; Munoz et al., 2017). Next to others, the somatostatin-expressing non-martinotti cells include basket cells (Ma et al., 2006). In contrast to martinotti cells, a subset of somatostatin-positive basket cells is also associated with the expression of parvalbumin (Nassar et al., 2015). The axonal projections of basket cells have a tendency to stay within a particular cortical layer, enabling them to extensively spread out and reach the majority of nearby pyramidal neurons (Packer and Yuste, 2011). Another important type of parvalbumin-expressing interneuron are chandelier cells (DeFelipe and Gonzalez-Albo, 1998). However, these cells do not express somatostatin. Chandelier cells innervate pyramidal cells at their axon initial segment (Somogyi, 1977). Moreover, similar to parvalbumin-expressing basket cells, parvalbumin-expressing chandelier cells exhibit fast-spiking characteristics that enable them to modulate neural activity and coordinate synchronized neural oscillations (Gulyas et al., 2010; Povysheva et al., 2013; Inan and Anderson, 2014). The remaining 30% of the interneuron population are formed by 5-hydroxytryptamine 3a receptor (5HT3_a_R)-expressing cells which constitute a very heterogenous group (Lee et al., 2010; Tremblay et al., 2016). In general, the 5HT3_a_R-expressing interneurons can be subdivided into VIP and non-VIP cells. The percentual amount of parvalbumin-, somatostatin- and 5HT3_a_R-expressing interneurons varies depending on the cortical layer (Rudy et al., 2011).

### 1.3. Fate and maturation of cortical interneuron progenitors

The fate of interneuron progenitors strictly depends on their origin (medial ganglionic eminence, caudal ganglionic eminence, and embryonic preoptic area) and consequently on their transcriptional programs (Kelsom and Lu, 2013; Kessaris et al., 2014; Lim et al., 2018). The transcriptional regulation of the interneuron differentiation and maturation is highly complex and will not be further themed in this review. Briefly, interneurons that originate from the medial ganglionic eminence differentiate into parvalbumin- and somatostatin-positive interneurons (Xu et al., 2005; Kelsom and Lu, 2013; Lim et al., 2018). Here, sonic hedgehog (Shh) signaling acts on the transcription factor Nkx2 homeobox 1 (Nkx2.1) which in turn activates Lim Homeobox 6 (Lhx6) and 8 (Lhx8) downstream (Liodis et al., 2007; Du et al., 2008; Zhao et al., 2008; Xu et al., 2010; Flandin et al., 2011; Kelsom and Lu, 2013). The activation of the transcription factors drives the differentiation to parvalbumin- and somatostatin-expressing subpopulations. Furthermore, members of the Dlx-family and aristaless related homeobox (Arx) contribute to the differentiation of parvalbumin-expressing interneurons (Cobos et al., 2005). In contrast, interneuron progenitors deriving from the caudal ganglionic eminence mainly give rise to 5HT3_a_R-expressing interneurons in the cortex and utilize Gs Homeobox 1 (Gsx1), 2 (Gsx2), Mash-1 and furthermore members of the Dlx-family in the later downstream signaling during their differentiation (Casarosa et al., 1999; Xu et al., 2010). Finally, progenitors of the preoptic area show an expression of Nkx2.1 and Nk5 homeobox 1 (Nkx5.1) but not Lhx6 turning them different in comparison to progenitors of the medial ganglionic eminence (Flames et al., 2007; Gelman et al., 2009). After the migration and differentiation interneuron progenitors of the preoptic area contribute to neuropeptide Y (NPY)- and reelin-expressing inter-neurons that are mainly located in the superficial layers of the cortex (Gelman et al., 2009). In contrast to intrinsic transcription factors that determine the fate of interneuron subtypes in the cortex, the orthodenticle homeobox protein 2 (OTX2) acts as a paracrine signaling molecule that is bound by so-called perineuronal nets (PNNs) which represent a specialized and dense form of the extracellular matrix (ECM; Beurdeley et al., 2012). In the cortex, OTX2 is captured by parvalbumin-expressing neurons during postnatal development and closes the critical phase (Sugiyama et al., 2008). The interactions between OTX2, parvalbumin-expressing neurons and PNNs will be described in more detail in a later paragraph. It is interesting to note that during early neural development, OTX2 plays a crucial role in the specification of various neuronal subtypes, including midbrain dopaminergic neurons and thalamic GABAergic interneurons (Vernay et al., 2005; Golding et al., 2014; Panman et al., 2014). Interestingly, a conditional knockout of OTX2 in the telencephalon leads to abnormalities in neurogenesis and oligodendrogenesis in the rostral telencephalon and the medial ganglionic eminence. In contrast, parvalbumin-expressing neurons in the cortex of these animals showed no significant changes in their number (Hoch et al., 2015). The OTX2-mediated maturation of cortical parvalbumin-expressing neurons appears to follow a different mechanism than that of OTX2-mediated maturation of midbrain neurons. In the cortex, the extracellular matrix in the form of PNNs seems to play a significant role in this process.

## 2. The extracellular matrix and its impact on parvalbumin-expressing interneurons

### 2.1. Perineuronal nets – A specialized ECM structure around parvalbumin-expressing interneurons

The ECM is a non-cellular network of secreted molecules, which can provide structural, but also biochemical support to the tissue. Furthermore, it is an important regulator of cellular processes like apoptosis, proliferation, differentiation, synaptogenesis and many more (Adams and Watt, 1993; Frisch and Francis, 1994; Dityatev et al., 2006; Schaefer and Schaefer, 2010). Based on bioinformatic analysis about 300 genes coding for glycoproteins or proteoglycan cores have collectively been designated as pertaining to the matrisome and 500 further genes have been classified as associated compounds (Hynes and Naba, 2012; Naba et al., 2012). In the central nervous system (CNS), the ECM is divided into three compartments, namely the interstitial matrix, PNNs and the basement membrane. As in all tissues and organs, the ECM also surrounds the cells in the brain and, together with the interstitial fluid, makes up approximately 20% of the brain volume (Nicholson and Hrabetova, 2017).

The ECM molecules not only occur loosely as an interstitial matrix in the extracellular space, but also as highly organized, specialized ECM structures. One specialized cell-adherent form of the ECM is represented by the basement membrane (BM). The BM in the brain is an important component of the blood–brain barrier. In addition to brain microvascular endothelial cells, pericytes and astrocytes, the BM represents the non-cellular component of the blood–brain barrier (Persidsky et al., 2006). However, the BM does not occur in the cellular environment of parvalbumin-expressing neurons and is therefore of no further concern for this review.

**Figure 1:**
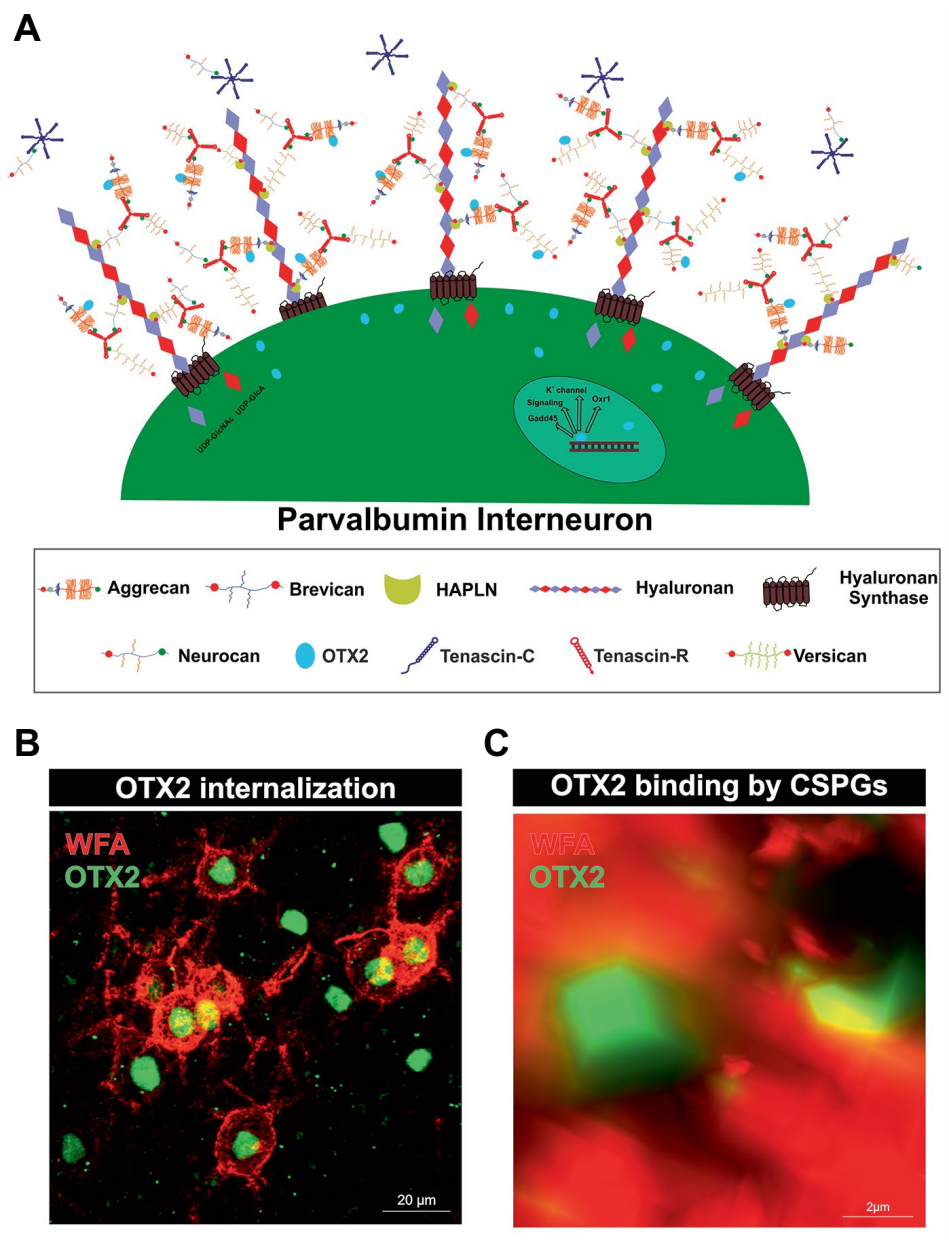
Internalization of OTX2 by Parvalbumin interneurons mediated by PNNs. **(A)** Scheme of PNN surrounding a parvalbumin interneuron. PNNs consist of a hyaluronan backbone, which interacts with lecticans and link proteins of the hyaluronan and proteoglycan binding link protein family (HAPLN) and forms the ternary complex. The lecticans aggrecan, brevican, neurocan, and versican, in turn can bind trimeric tenascin-R to their G3 domain and form aggregates. Glycosaminoglycan chains of the lecticans can bind the transcription factor OTX2 and present it to the cell surface, where it is then internalized. Inside the cell OTX2 can activate transcriptional activity of genes such as *Oxr1*, K_V_3.1, or *GAD45* and promote parvalbumin cell maturation. **(B)** Image of an immunohistochemical staining of PNNs (red) and OTX2 (green) in the murine cerebral cortex recorded by structured illumination microscopy. PNNs were visualized by lectin labeling of the CS-GAGs *via wisteria floribunda* agglutinin (WFA). Most of the OTX2-positive cells are PNN-enwrapped, indicating the important role of the PNN-OTX2 binding for internalization. **(C)** Higher magnification shows the close proximity between the WFA- and OTX2-positive signal. WFA marks the CS-chains of the proteoglycans, which also act as binding site for OTX2.

Rather, there is another specialized type of ECM that forms around parvalbumin-expressing interneurons and plays an important role in their maturation and synaptic organization, called PNNs. They arise at the cellular surface. There, the enzyme hyaluronan synthase (HAS) synthesizes pericellular hyaluronan and retains it on the cell surface (Toole, 2004). Link proteins of the hyaluronan and proteoglycan binding link protein family (HAPLN) can bind to the hyaluronan “backbone” and besides to the N-terminal globular (G1) domain of the lecticans (Perin et al., 1987). Interactions between hyaluronan, HAPLNs and lecticans leads to the formation of a ternary complex (Heinegard and Hascall, 1974; Matsumoto et al., 2003; Seyfried et al., 2005). These aggregates form the basic structure of PNNs. The lecticans are a family of chondroitin sulfate proteoglycans (CSPGs) and include the four members aggrecan, brevican, neurocan and versican (Yamaguchi, 2000; Pacharra et al., 2013). In addition, distinct lecticans can bind to the fibronectin type III repeats of tenascin-R (TNR) or tenascin-C (TNC) *via* their C-terminal G3 domain (Rauch et al., 1997; Lundell et al., 2004). Linked by disulfide bridges TNR can build trimeric structures and therefore has multiple binding-sites for the lecticans, which in turn expands the net structure (Figure 1A; Pesheva et al., 1989). HAS, HAPLNs and aggrecan play a particularly important role in PNN formation as cells lacking one of these molecules are incapable to build regular PNNs (Kwok et al., 2010). Although the basic structure of the PNNs is always the same, PNN composition is heterogeneous with regard to the enveloped cell type and the brain area in which the PNN is located. The composition of PNNs can even vary within the same brain region and among the same neuron types. Studies utilizing monoclonal antibodies Cat-301, Cat-315, and Cat-316, which label different glycosylation patterns of aggrecan, demonstrate that neurons themselves regulate their ECM composition (Matthews et al., 2002). The different glycosylation patterns of aggrecan indicate different interaction properties with other molecules. Co-localization with OTX2 is observed in aggrecan positive for Cat-316, while aggrecan positive for WFA is OTX2 negative, suggesting that PNN heterogeneity has a role in the maturation of parvalbumin positive neurons by selective binding of OTX2 (Miyata et al., 2018). In addition, aggrecan as well as brevican and TNR exhibit distinct distribution patterns within PNNs in different brain regions (Dauth et al., 2016). However, the impact of the diverse composition of PNNs on their functionality remains inadequately investigated.

PNNs mainly envelop parvalbumin expressing, fast-spiking interneurons. There are descriptions of PNN-enwrapped interneurons positive for parvalbumin in the cerebral cortex, amygdala, hippocampus, and cerebellum (Hartig et al., 1995, 1999; Yamada et al., 2015). However, the bulk of knowledge concerning the interplay of parvalbumin-expressing interneurons and their surrounding PNNs has been derived from *in vivo* investigations conducted within the neocortex. Thereby, it was demonstrated that treatment with ChABC in the visual cortex resulted in a reduction of PNNs, which in turn was accompanied by a significant decrease in the mean spiking activity of parvalbumin-expressing neurons (Lensjo et al., 2017). Also, inhibition of neurons expressing parvalbumin can lead to regression of their PNNs. This suggests that individual parvalbumin-expressing neurons may be capable of regulating their own PNN density (Devienne et al., 2021). PNNs ensure activity of parvalbumin-expressing interneurons but also exert a protective effect. Fast-spiking parvalbumin-expressing interneurons require rapid cation exchange for their function. Enveloping PNNs with their negatively charged CSPGs can attract and sequester cations from the parvalbumin-expressing neurons (Hartig et al., 1999). The negative charge of PNNs can also protect parvalbumin-expressing interneurons from oxidative stress, as these are particularly susceptible to it due to their high energy consumption (Suttkus et al., 2014). In fact, not all parvalbumin-expressing interneurons are surrounded by a PNN, as a small subpopulation of parvalbumin-expressing interneurons does not appear to form them (Yamada et al., 2015). The exact mechanisms that determine whether an interneuron forms a PNN or remains uncovered are not yet known. One important factor for the maturation of PNNs and the neurons they surround is sensory experience. Therefore, dark rearing of mice leads to a reduced density of PNNs in the visual cortex and whisker trimming results in a specific decrease of PNNs around parvalbumin expressing interneurons in the barrel cortex (McRae et al., 2007; Ye and Miao, 2013). Nonetheless, the formation of PNNs around parvalbumin-expressing interneurons is considered to be an essential step in their maturation and survival. In conclusion, PNNs are a specialized ECM structure that forms mainly around parvalbumin-expressing interneurons in the CNS. They play an important role in the maturation and synaptic organization of these interneurons. PNNs also ensure the activity of parvalbumin-expressing interneurons and have a protective effect. Hence, the interplay between PNNs and parvalbumin-expressing interneurons is crucial for proper brain function.

### 2.2. Parvalbumin interneuron maturation and PNN formation as regulator of neuronal plasticity

During development, many brain areas show a phase of increased plasticity in response to sensory stimuli (Diamond et al., 1993; Knudsen, 1998; Kannan et al., 2016). This was first demonstrated by monocular deprivation of cats, which resulted in a shift in the ocular dominance columns towards the non-deprived eye in the visual cortex (Wiesel and Hubel, 1963). These critical periods allow the neuronal circuitry to optimally adapt to the incoming sensory information. Decisive for the onset of the critical period is the maturation of inhibitory GABAergic circuits. This was also shown in the visual cortex of a mouse model with a gene-targeted disruption of glutamic acid decarboxylase, which is involved in the synthesis of GABA. These animals do not react to monocular deprivation with a shifted ocular dominance since the lack of inhibition by GABA leads to an absence of plasticity. In turn, treatment of these animals with benzodiazepines, a GABA agonist, restored intracortical inhibitory transmission and plasticity (Hensch et al., 1998).

In this context, parvalbumin-expressing neurons play a particularly important role for the critical period, as they inhibit excitatory circuits and modulate synchronized neuron activity (Kubota, 2014). The formation of PNNs around parvalbumin-expressing cells acts as a molecular brake for the critical period in the cortex. It could be shown that experimental decomposition in adult animals reopens the plastic period. Pizzorusso and his colleagues showed that digestion of CSPGs with chondroitinase-ABC (ChABC) in the adult rat visual cortex could reactivate cortical plasticity (Pizzorusso et al., 2002). In the same vein, HAPLN1-deficient mice display impaired PNN formation, resulting in juvenile levels of ocular dominance plasticity (Carulli et al., 2010). But how does PNN formation initiate the end of the critical period? On the one hand, the CSPGs of the PNNs exert an inhibitory effect on axonal sprouting. Therefore, enzymatic digestion of CSPGs with ChABC in the spinal cord after injury facilitates the regeneration of ascending sensory projections and descending corticospinal tract axons. This contributes to functional recovery of locomotor and proprioceptive behaviors (Bradbury et al., 2002). The sulphation pattern of the GAGs is important for the axonal guidance and the regulation of synaptogenesis by CSPGs. *In vitro* studies with cultured primary neurons document that chondroitin-4-sulfate (CS-A) but not chondroitin-6-sulfate acts in an inhibitory manner on growing axons (Wang et al., 2008). Along these lines, disruption of CSPGs by ChABC favors the formation of synapses on hippocampal neurons *in vitro* (Pyka et al., 2011).

On the other hand, PNNs can bind recognition molecules and present these to the PNN-enwrapped neuron and their environment, which may trigger intrinsic cellular processes and have an impact on neuronal plasticity. One example is the chemorepulsive axon guidance molecule semaphorin 3A (Sema3A). The sulphation of CS-GAG chains is crucial, as chondroitin-4-6-sulfate (CS-E) has been deciphered as critical element of the binding site for Sema3A (Dick et al., 2013). In the cortex the Sema3A receptor components PlexinA1 and A4 are selectively expressed by inhibitory neurons encased by PNNs (Vo et al., 2013). Presentation of Sema3A by PNNs facilitates its binding by a receptor complex consisting of neuropilin-1, a member of the plexin family, and cell adhesion protein L1 (He and Tessier-Lavigne, 1997; Takahashi et al., 1999; Castellani et al., 2000). Binding of Sema3A to the receptor complex leads to growth cone collapse caused by depolymerizing actin filaments in response to the activation of members of the RhoA-family of small GTPases (Vastrik et al., 1999; Mikule et al., 2002; Zanata et al., 2002; Stankiewicz and Linseman, 2014). Thereby, Sema3A may contribute to the restraint of the projection of axons and decrease plasticity, an important step for the closure of the critical period (Boggio et al., 2019).

OTX2 is another molecule of interest that attaches to PNNs and is presented to net-wearing cells. OTX2 is crucial for parvalbumin-expressing neuron maturation and PNN formation and will be discussed in more detail in the following paragraph.

Taken together, critical periods allow neuronal circuits to optimally adapt to incoming sensory information. The maturation of inhibitory GABAergic circuits is crucial for the onset of these periods, and parvalbumin-expressing neurons play a significant role in inhibiting excitatory circuits and modulating synchronized neuron activity. The formation of PNNs around these interneurons functions as a molecular brake of the critical period in the cortex, inhibiting axonal sprouting and binding regulatory molecules such as Sema3A and OTX2 to impact intrinsic cellular processes and decrease plasticity.

### 2.3. OTX2 internalization by parvalbumin cells and activation of transcriptome activity

Similar to Sema3A, OTX2 binds with high affinity to CS-E of PNNs (Figures 1B,C). For this, OTX2 possesses a specific binding site containing an arginine-lysine doublet (RK peptide). Infusion of the RK peptide reduces parvalbumin and PNN expression and reopens plasticity in the visual cortex (Beurdeley et al., 2012). Interestingly, it has been shown that the binding and internalization of OTX2 contribute to the maturation of parvalbumin-expressing interneurons (Beurdeley et al., 2012; Bernard and Prochiantz, 2016; Lee et al., 2017; Sakai et al., 2017). OTX2 is not dependent on an energy-requiring carrier to traverse the cell membrane, but probably uses the organization of a micelle structure (Edelstein and Smythies, 2013). Inside the cell OTX2 can transit to the nucleus and activate transcription. Using chromatin immunoprecipitation sequencing in the juvenile mouse cortex, genome-wide binding sites of OTX2 were identified. Next to circadian rhythm genes including *Clock*, expression of potassium channels belonging to the K_v_3.1-family and the *oxidative resistance 1* (*Oxr1*) were induced by OTX2 (Sakai et al., 2017). The K_v_3.1 gene is tightly regulated in the juvenile cortex, indicating maturation conferring properties of OTX2 for parvalbumin-expressing neurons. This voltage-dependent channel regulates inhibitory function of parvalbumin-positive neurons in the neocortex, and its disruption broadens action potentials while reducing net inhibitory function, leading to slowed input loss from a deprived eye and altered critical period plasticity, which bears implications for mental illnesses (Matsuda et al., 2021). *Oxr1* expression is thought to be important for the protection of the parvalbumin-expressing interneurons from oxidative stress. Other genes containing a binding site for OTX2 are the *Gadd45b/g* genes. Interestingly, augmented expression of these genes mediated by OTX2 correlates with an increase in the expression of plasticity genes in the visual cortex (Apulei et al., 2019).

It is also suspected that OTX2 internalization induces the expression of PNN components, as infusion of OTX2 in the visual cortex increases the number of PNNs around parvalbumin-expressing neurons (Sugiyama et al., 2008). Furthermore, the knockdown of *OTX2* in the choroid plexus decreases parvalbumin expression and PNN assembly (Spatazza et al., 2013).

Although other GABAergic interneurons can internalize OTX2 as well, over 70% are parvalbumin-expressing (Sugiyama et al., 2008). OTX2 does not originate in the cortex, but rather stems from two other regions where OTX2 is synthesized. It has to be transferred from these sites into the cortex. One source are photoreceptors and bipolar cells of the retina, the other source are epithelial cells of the choroid plexus (Nishida et al., 2003; Koike et al., 2007; Spatazza et al., 2013; Kaufman et al., 2021). OTX2 synthesized in the retina is transported along the retinal ganglion cell axons to the lateral geniculate nucleus. From there neurons that project to the visual cortex shuttle it *via* their axons (Sugiyama et al., 2008; Torero Ibad et al., 2020).

The ECM also appears to intervene in the transfer of OTX2 from the retina to the visual cortex. Previous studies of our laboratory showed that mice deficient for the four ECM molecules brevican, neurocan, tenascin-C and -R had reduced numbers of OTX2-positive cells in the visual cortex. In contrast, other cerebral cortex areas showed comparable levels of OTX2 both in the knockout and in the wildtype. In addition, the number of parvalbumin-expressing interneurons appeared reduced in the visual cortex, but not in other cortical areas. In contrast, PNN numbers were diminished in all cortical areas examined (Mueller-Buehl et al., 2022).

The precise mechanisms of OTX2 transport have not been investigated in this mouse model. A possible explanation for the OTX2 reduction could be a disrupted axonal transport from the retina to the visual cortex, as all the four deleted molecules are relevant for axonal pathfinding. The CSPGs Bevican and neurocan exert inhibitory effects on axons, as described above (Fawcett, 2015). CSPGs are well known to influence axonal pathfinding along visual pathways. As an example, addition of exogenous chondroitin sulfate results in the disturbed trajectory of retinal axon within the optic tract (Walz et al., 2002). Tenascins can also influence axonal guidance and sprouting (Faissner, 1997). Axon pathfinding in the developing optic system of zebrafish is dependent on regular TNR expression. Reducing the expression of TNR by injecting morpholinos into fertilized eggs leads to enhanced axon branching along the optic tract (Becker et al., 2003). TNC can promote, but also inhibit neurite outgrowth and cultivated rat retinae exposed to TNC reveal increased fiber length. So TNC appears to promote axon growth activity in some situations (Siddiqui et al., 2008). Disturbed axonal sprouting could also affect higher visual areas, as molecular signals conveyed by sensory visual afferents which promote maturation of cortical circuits might not reach their target region. It is possible that a disturbance in axonal transport of OTX2 in quadruple knockout mice along the visual pathways could impact the levels of OTX2 in the visual cortex. OTX2 in the visual cortex might primarily originate from the retina, while other cortical regions in the adult cortex could receive OTX2 from the choroid plexus. In agreement with this interpretation, the mutant animals exhibit normal distribution of OTX2 in these areas.

In summary, the ECM provides an environmental milieu that protects and promotes the maturation of parvalbumin-expressing cells.

## 3. PNNs and parvalbumin-expressing interneurons as regulators of the synaptic E/I balance

**Figure 2:**
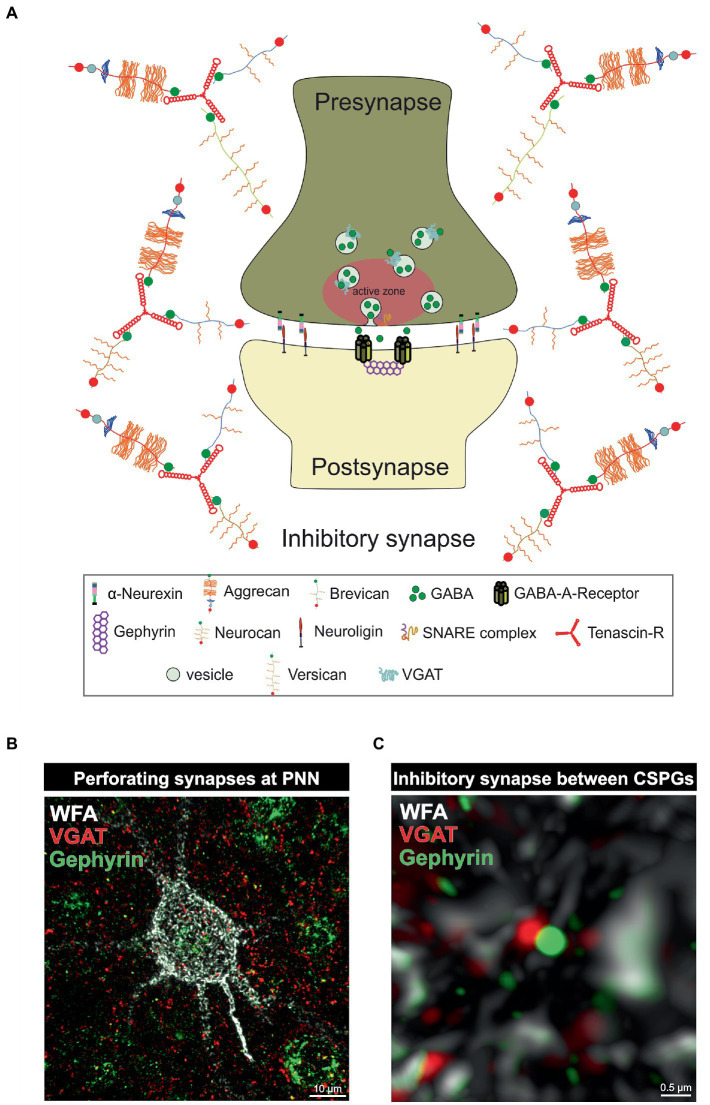
PNNs as organizer of inhibitory synapses. **(A)** Scheme of an inhibitory GABAergic synapse surrounded by a PNN. PNNs surround pre- and postsynapse and stabilize the neuronal connection. Afferent axons can perforate the PNN “wholes” and build synaptic boutons with postsynapses at the cell surface. Components of the PNNs can reach the perisynaptic space and limit synaptic receptor mobility. They also act as buffer and can supply the synapse with cations important for synaptic function. **(B)** Image of an immunohistochemical staining of a PNN and perforating inhibitory synapses recorded by structured illumination microscopy. PNNs were visualized by lectin labeling of the CS-GAGs *via* WFA (white), inhibitory presynaptic puncta *via* an antibody against VGAT (red) and inhibitory postsynaptic puncta *via* an antibody against gephyrin (green). **(C)** Pre- and postsynaptic inhibitory elements use the space between the PNN components of the PNNs to form inhibitory synapses there, as shown at the higher magnification. The close proximity to the PNNs ensures synaptic integrity.

Parvalbumin-expressing interneurons are discussed to have a critical role in regulating the E/I balance, as they regulate pyramidal neuron activity (Ferguson and Gao, 2018). An impaired excitatory/inhibitory ratio can contribute to severe pathological conditions, as discussed in the further course of this review. On the molecular level the sensitive E/I balance is preserved through precise synaptic connections between inhibitory and excitatory neurons. An important regulator for synaptic stabilization and modulation is the ECM (Faissner et al., 2010). For instance, CSPG digestion on murine organotypic hippocampal slices leads to enhanced motility of dendritic spines and microinjection of ChABC near dendritic segments induces spine remodeling independently of PNNs (Orlando et al., 2012). In addition, ECM molecules can interact with transmembrane proteins and affect synaptic structure (Dzyubenko et al., 2016). Aggrecan as an example can interfere with integrin signaling and therefore inhibit axon growth of adult rat dorsal root ganglia (Tan et al., 2011). Furthermore, ECM components can affect synaptic vesicle release. Reelin can enhance spontaneous neurotransmitter release at the presynapse and laminins organize the synaptic vesicle release at the active zone (Bal et al., 2013; Rogers and Nishimune, 2017). An extensive review of the synaptic ECM and its role in the modulation and functionality of synapses has already been given (Dankovich and Rizzoli, 2022). Due to the significant alterations of PNNs and the associated changes in synaptic structures in neuropsychiatric disorders, this specialized form of ECM is of particular interest to the scientific community. PNNs have a strong influence on synapse formation and function of neurons they surround (Figure 2). Synaptic inputs can perforate the “wholes” of PNNs, as shown by super-resolution microscopy (Sigal et al., 2019). Through the negative charge of the PNN CSPGs and hyaluronan they can buffer cations and provide the synapse with cations for synaptic transmission (Hartig et al., 1999). This PNN feature is especially important for fast-spiking parvalbumin-expressing neurons as they are highly active and therefore have a strong demand for cations. Impairments of the PNN structure can strongly disturb the integrity of synapses adjoined to them. As described above PNNs furthermore inhibit neurite growth through the inhibitory properties of their CSPGs and through the binding of chemo repulsive molecules. In addition, PNN components can directly interact with synaptic structures. TNR can bind GABA receptors and hyaluronan modulates postsynaptic L-type calcium channels (Bukalo et al., 2007; Kochlamazashvili et al., 2010). PNNs also regulate the lateral diffusion of α-amino-3-hydroxy-5-methyl-4-isoxazolepropionic acid (AMPA) receptors. Enzymatic digestion of PNNs by hyaluronidase leads to an increased AMPA receptor mobility at the neuronal surface (Frischknecht et al., 2009). In this context, it is not surprising that PNN impairments lead to changes in the synapses on the neurons they encase. Also, the deletion of single PNN components can affect synaptic integrity. TNC and TNR as well as brevican single knockout mice reduced long term potential (LTP; Saghatelyan et al., 2001; Brakebusch et al., 2002; Evers et al., 2002). *In vitro* analyses of primary hippocampal neurons of mice lacking brevican, neurocan, TNC and TNR, called quadruple knockout mice, revealed severe deficits in PNN formation and synapse formation was impaired (Rauch et al., 2005; Geissler et al., 2013). Further *in vitro* studies on hippocampal neurons of quadruple knockout mice showed, that the loss of the four ECM molecules leads to an increase in the number of excitatory, but a decrease in the number of inhibitory synaptic molecules. This synaptic E/I imbalance results in an increased neuronal network activity as shown by multielectrode array measurements (Gottschling et al., 2019). Recent studies of our laboratory revealed a similar synaptic E/I imbalance along quadruple knockout cortical PNNs *in vivo*. Here, the quadruple knockout not only caused a loss of PNNs, but also the structure of the residual PNNs was strongly disturbed. Analyses of synaptic molecules on the remaining PNNs pointed to an increased number of excitatory, but a reduced number of inhibitory synaptic elements (Mueller-Buehl et al., 2022). However, the consequences for neuronal functionality or synaptic transmission were not examined in this study. It should be noted that not only PNNs, but also large portions of the neural ECM are composed of a hyaluronan-HAPLN backbone coupled with CSPGs, which are in turn connected to each other *via* TNR. PNNs represent a particularly condensed form of this ECM (Dityatev and Schachner, 2003). Therefore, the consequences of enzymatic digestion or genetic knockouts of ECM components cannot exclusively be attributed to PNNs, as these interventions also affect the interstitial ECM. Still, in the light of its apparent disturbances and discrete phenotypes with reference to distinct physiological parameters the quadruple knockout mouse model appears attractive for behavioral studies in the future. Taken together, the present data underline the importance of the matrisome, and in particular of its condensed PNN variant for the tuning of the synaptic E/I balance. Since disorders of the E/I balance ultimately lead to abnormal behavior and accompany a wide range of neurodegenerative and psychiatric diseases, the ECM is progressively perceived as a novel target for potential therapeutic interventions.

## 4. The influence of the extracellular matrix on parvalbumin-expressing neurons in disease

### 4.1. Role of PNN-carrying interneurons for the neuropsychiatric disorder schizophrenia

**Figure 3:**
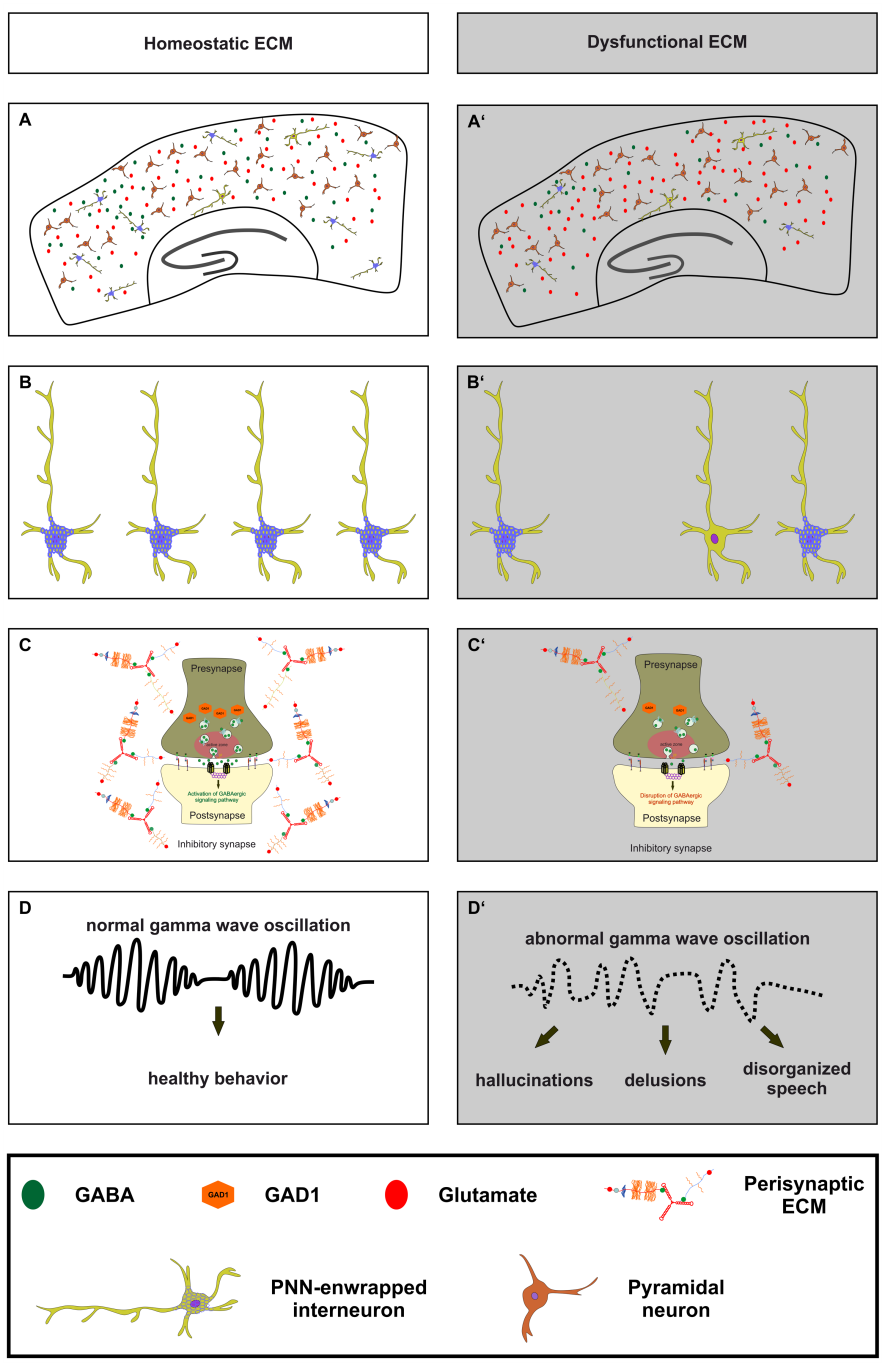
The importance of a homeostatic ECM for parvalbumin-expressing interneurons, synaptic transmission and ultimately healthy behavior. **(A,A’)** An illustration of a homeostatic ECM in the cortex with regulated E/I balance, where PNN enwrapped parvalbumin interneurons (light green with blue nets) and a few not PNN enwrapped parvalbumin interneurons are depicted. Pyramidal cells (dark red) and a balanced neurotransmitter release (GABA as green circles and glutamate as red circles) are also shown. In contrast, **(A’)** shows dysfunctional ECM in the cortex, where the number of PNNs and their enwrapped parvalbumin interneurons is reduced, and a disturbed E/I balance is indicated by reduced GABA and increased glutamate levels. **(B,B’)** In comparison to the homeostatic ECM, the dysfunctional ECM shows a lower number of parvalbumin interneurons and a reduced level of PNNs around these cells. **(C,C’)** At the synaptic level, a dysfunctional ECM in neuropsychiatric diseases like schizophrenia can lead to reduced levels of GAD1 (orange hexagons), which in turn leads to a disruption of GABAergic signaling pathways. The altered ECM in the perisynaptic region is also depicted. Since the ECM has been described as an important regulator of synaptic integrity, changes in its composition may be associated with altered GABAergic synaptic transmission. **(D,D’)** Ultimately, the impairments of the ECM, parvalbumin-expressing neurons, and synaptic E/I balance result in abnormal gamma wave oscillations in neuropsychiatric diseases. This, in turn, culminates in abnormal behavior, manifested as hallucinations, delusions, and disorganized speech.

As this review addresses the role of the neural matrisome and its impact on the E/I-balance it is important to consider pathological situations in which the E/I-balance is disrupted (Figure 3). For this reason, the following paragraph focuses on the neuropsychiatric disease schizophrenia and the role of GABAergic interneurons for the cognitive symptoms of this disease (Lewis et al., 1999; de Jonge et al., 2017). Previous studies revealed that patients suffering from schizophrenia show reduced *GAD1* expression levels, however, without a significant loss of GABAergic interneurons (Akbarian et al., 1995; Guidotti et al., 2000; Addington et al., 2005; Mitchell et al., 2015). Furthermore, an extensive *postmortem* transcriptional profiling study proved a significant reduction of signaling pathways associated with GABA-receptor signaling (Lanz et al., 2019). Additional studies identified subtype-specific disruptions of parvalbumin-expressing interneurons including basket cells (Curley and Lewis, 2012; Glausier et al., 2014; Fish et al., 2021) and chandelier cells (Woo et al., 1998; Pierri et al., 1999; Lewis, 2011) as well as of somatostatin-expressing interneurons including martinotti cells and non-martinotti cells (Lewis et al., 2008; Van Derveer et al., 2021). Less is known about the role of 5HT3_a_R-expressing interneurons in schizophrenia. However, the implication of this interneuron-subtype was discussed in the context of the serotonin hypothesis of schizophrenia (Engel et al., 2013).

On a functional level schizophrenic patients show abnormal gamma wave oscillations in the cortex, as previous studies have shown (Gallinat et al., 2004; Lewis et al., 2005; Cho et al., 2006; Light et al., 2006; Ferrarelli et al., 2008; Spencer et al., 2008; Williams and Boksa, 2010; Yanagi et al., 2022). Furthermore, recently published findings unraveled that deficits in gamma wave oscillations are correlated with verbal learning and memory dysfunctions as well as with sensory gating deficits in schizophrenia patients (Nguyen et al., 2020; Tanaka-Koshiyama et al., 2020). Inhibitory gamma waves are associated with proper cognitive functions and of great interest since the cognitive symptoms of schizophrenia are strongly impairing the life quality of patients and cannot be treated adequately (Fell et al., 2001; Lutzenberger et al., 2002; Gruber et al., 2004; Fries, 2009; Bosman et al., 2014). Gamma wave oscillations are generated by parvalbumin- and somatostatin-expressing interneurons in the cortex and in the hippocampus (Veit et al., 2017; Espinosa et al., 2019; Kawaguchi et al., 2019; Antonoudiou et al., 2020; Kriener et al., 2022). Interestingly, it has been observed that N-methyl-D-aspartate receptors (NMDARs) on parvalbumin-expressing interneurons have a critical function for gamma wave generation (Carlen et al., 2012). A specific depletion of NMDARs on parvalbumin-expressing interneurons resulted in altered gamma band properties and an impaired hippocampal synchrony, as well as with deficits in spatial representations and working memory (Korotkova et al., 2010). This aspect is of great interest since NMDAR-antagonists like ketamine or phencyclidine and cases of autoimmune NMDAR encephalitis induce symptoms associated with schizophrenia (Javitt and Zukin, 1991; Lahti et al., 1995, 2001; Omdal et al., 2005; Kayser and Dalmau, 2016; Beck et al., 2020).

Based on these observations a proper functionality of receptors on the surface of parvalbumin-expressing interneurons is crucial for the generation and maintenance of gamma waves. Furthermore, a synaptic E/I-balance on parvalbumin-expressing interneurons is important for the electro-physiological properties and consequently for the firing behavior of excitatory projection neurons contacted by inhibitory interneurons. Here, PNNs were identified as important regulators of the synaptic stability and E/I-balance, as previously described in this review article (Frischknecht et al., 2009; Pyka et al., 2011; Gottschling et al., 2019; Wegrzyn et al., 2021a; Mueller-Buehl et al., 2022). Regarding schizophrenia, several studies revealed significantly reduced or weaker immunostained PNNs in the cortex and amygdala of *postmortem* schizophrenic patients (Pantazopoulos et al., 2010; Mauney et al., 2013; Pantazopoulos et al., 2015; Enwright et al., 2016). Therefore, disruptions of PNNs in schizophrenic patients might affect the synaptic balance on parvalbumin-expressing interneurons and their electrophysiological properties as well as cortical gamma oscillations. Importantly, a recent study confirmed this assumption and revealed that a digestion of PNNs *via* the stereotactical administration of chondroitinase ABC induced a decrease of inhibitory synaptic puncta on the perisomatic region and a decrease of the gamma-activity in the medial prefrontal cortex of mice (Carceller et al., 2020). Similarly, in hippocampal networks *in vitro* the depletion of the ECM resulted in a reduction of inhibitory connectivity (Dzyubenko et al., 2021). Another study could document altered gamma oscillations when PNNs were digested (Lensjo et al., 2017).

Although there is growing evidence of PNN and interneuron deficits in schizophrenia patients and animal models for schizophrenia, less is known about the mechanisms how the disturbances manifest. Both the formation of PNNs and the expression of parvalbumin are activity-dependent mechanisms (Patz et al., 2004; Dityatev et al., 2007). Therefore, a reduced activation of parvalbumin-expressing interneurons during development might provide a possible explanation for the above-mentioned deficits. Here, hypofunctional NMDARs on the surface of GABAergic interneurons could hypothetically be responsible for the reduced activation and a delayed maturation of interneurons in schizophrenia patients. It has been reported that the glutamatergic neurotransmission *via* NR2A-containing NMDARs on parvalbumin-expressing interneurons in the cortex may be altered in schizophrenia (Bitanihirwe et al., 2009). With regard to this aspect, a recently published study demonstrated that an early interneuron-specific NMDAR ablation induces a functional hypoconnectivity of the prefrontal cortex with the hippocampus after adolescence in mice, and further schizophrenia-related characteristics (Alvarez et al., 2020). A second possible explanation might be that inflammatory processes and the associated activation of microglia alter PNN integrity. On the one hand, the evidence is rising that inflammatory processes occur in the CNS of schizophrenic patients (Bayer et al., 1999; Busse et al., 2012; Hwang et al., 2013; Lesh et al., 2018; Lanz et al., 2019). On the other hand several recent studies observed important regulatory functions of microglia for PNNs (Crapser et al., 2021; Venturino et al., 2021; Wegrzyn et al., 2021a,b). Inflammation and dyshomeostatic microglia might therefore disrupt PNNs and change the synaptic balance as well as interneuron properties.

Taken together, the evidence suggests that disruptions in the E/I-balance in schizophrenia plays a significant role regarding the cognitive symptoms of the disease. Studies have shown subtype-specific disruptions of GABAergic interneurons, particularly those expressing parvalbumin and somatostatin, which generate inhibitory gamma waves that are essential for proper cognitive functioning. NMDARs on parvalbumin-expressing interneurons also play a critical role in gamma wave generation, and proper functionality of receptors on their surface is crucial. PNNs have been identified as important regulators of the synaptic stability and E/I-balance, and disruptions of PNNs in schizophrenic patients might affect the synaptic balance on parvalbumin-expressing interneurons and their electrophysiological properties as well as cortical gamma oscillations.

### 4.2. Role of PNN-carrying interneurons in the neurodegenerative disease Alzheimer

**Figure 4:**
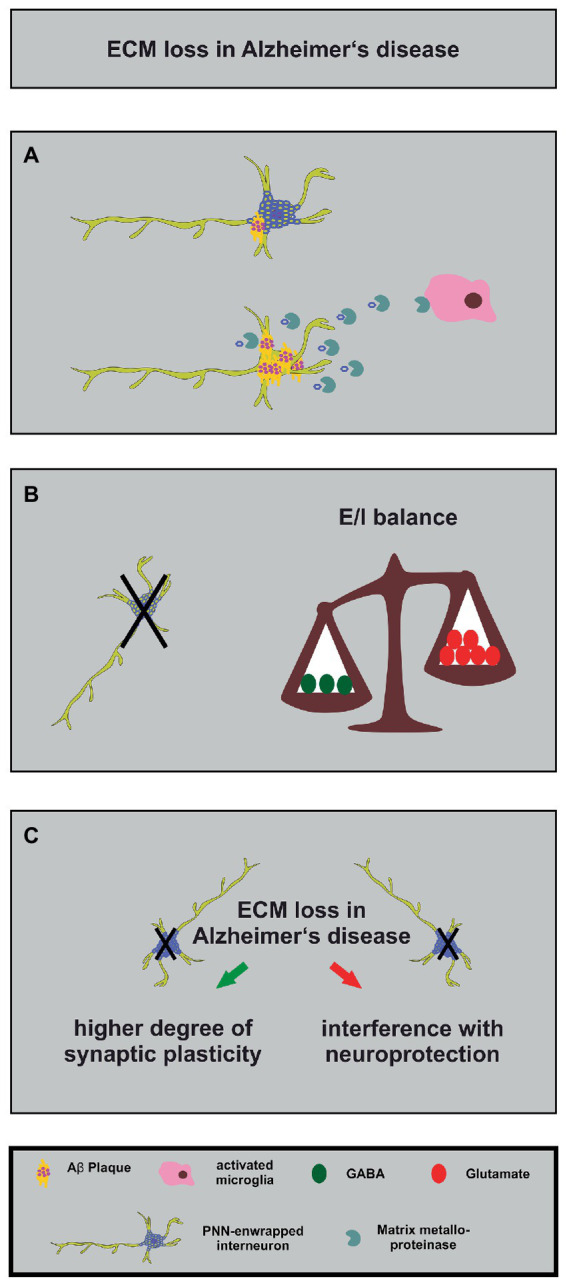
Alterations in the neuronal matrisome and its consequences in Alzheimer’s disease. **(A)** Degradation of PNN components by MMPs released by activated microglia (in pink) contributes to loss of neuroprotection of PNN-enwrapped interneurons (shown with mild Aβ-plaques), leading to increased Aβ-plaque accumulation and ultimately cell death in Alzheimer’s disease. **(B)** The loss of PNN-enwrapped interneurons (represented by a black cross) leads to a shift in the E/I balance towards increased excitation, as shown by the unbalanced scales. The left side of the scale, with fewer green circles representing GABA, is lower than the right side with more red circles representing glutamate, indicating a relative increase in excitatory signaling. This imbalance results from the loss of PNN-mediated neuroprotection, which highlights the critical role of the ECM in regulating neural circuit function in Alzheimer’s disease. **(C)** The ECM and interneuron loss in Alzheimer’s disease can lead, on the one hand, to increased synaptic plasticity (green arrow) as a compensatory mechanism for the loss of neuronal connections. On the other hand, it can interfere with PNN-mediated neuroprotection (red arrow). The loss of interneurons, in turn, can contribute to cognitive impairments.

Alzheimer disease is a neurodegenerative disorder which is the main cause of dementia, a condition characterized by a severe decay of cognitive functions (Calabro et al., 2021; Knopman et al., 2021; Trejo-Lopez et al., 2022). The extracellular accumulation of β-amyloid (Aβ) plaques and the presence of intracellular tau-containing neurofibrillary tangles were identified as the pathophysiological hallmarks of this disease (Glenner and Wong, 1984). Interestingly, the accumulation and the distribution of Aβ-plaques occurs in a predictable manner in five phases (Thal et al., 2002). A global meta-analysis unraveled that approximately 5–7% of the world’s population older than 60 years are affected by this disease and by its life-impairing symptoms (Prince et al., 2013). There is growing evidence of an E/I-imbalance that drives the pathology of Alzheimer disease (Palop et al., 2007; Sun et al., 2009; Bi et al., 2020; Lauterborn et al., 2021). This imbalance is characterized by a dysfunction of GABAergic interneurons accompanied by a shift towards excitation (Bi et al., 2020; Lauterborn et al., 2021). Interestingly, a recently published study utilized *postmortem* parietal cortex samples from middle-aged patients with an early-onset Alzheimer disease and verified elevated E/I ratios in this brain region (Lauterborn et al., 2021). Furthermore, it has been shown that the experimental overexpression of the human amyloid precursor protein (APP) in transgenic mice raised spontaneous as well as evoked excitatory currents while inhibitory currents were significantly reduced in acute hippocampal slices (Roberson et al., 2011). Since the regulation of the E/I-balance by the neural matrisome is in the focus of this review, the PNN-carrying subpopulation of GABAergic interneurons will be discussed in the context of Alzheimer disease in this paragraph (Figure 4).

PNNs seem to play different roles for several aspects of Alzheimer disease (Pantazopoulos and Berretta, 2016; Wen et al., 2018). Studies of *postmortem* human brains revealed a significant reduction of PNNs in the brain of Alzheimer disease patients (Kobayashi et al., 1989; Baig et al., 2005; Reichelt, 2020; Scarlett et al., 2022). Based on these findings and on the fact that PNNs stabilize synaptic contacts and reduce synaptic plasticity it was hypothesized that the loss of PNNs might serve as an endogenous compensatory mechanism which induces a higher grade of synaptic plasticity and counteracts the cognitive deficits of Alzheimer disease (Reichelt, 2020). This idea is supported by several studies that confirm the contribution of PNNs for memory functions and learning (Gogolla et al., 2009; Hylin et al., 2013; Romberg et al., 2013; Xue et al., 2014; Shi et al., 2019; Fawcett et al., 2022). In addition, direct injections of the ECM-degrading enzyme ChABC into the hippocampus of transgenic APP/PS1 mice restored on the one hand long-term potentiation properties and on the other hand the contextual memory performance of the animals (Vegh et al., 2014). Surprisingly, this study revealed an upregulation of ECM-molecules in the hippocampus which preceded the occurrence of Aβ-plaques (Vegh et al., 2014). Similar observations were reported for a transgenic mouse model which expresses a mutant P301S tau protein in neurons. Here, injections of ChABC into the perirhinal cortex could restore the object recognition memory 1 week post injection (Yang et al., 2015). In this context, it has to be considered that a treatment with ChABC does not solely act on PNNs but also digests the loose neural interstitial matrix, as already emphasized in a previous paragraph. In order to circumvent this side effect, antibodies that block the inhibitory 4-sulfated CSPG moieties which occur in PNNs have been used. Application of the antibodies restored the object recognition memory in the previously mentioned P301S mouse model for Alzheimer disease (Yang et al., 2017). The manipulation of the ECM has therefore been discussed as a possible treatment for the relief of the cognitive impairments. However, digesting or manipulating perineuronal nets might interfere with other PNN functions, as described below.

In addition to the plasticity-restrictive properties of PNNs, neuroprotection is another relevant function (Figure 4). Previous studies revealed that perineuronal nets act in a neuroprotective manner against iron-induced oxidative stress (Suttkus et al., 2012; Cabungcal et al., 2013). Furthermore, it has been shown that PNNs also protect against toxic Aβ-plaques in primary cortical neuron cultures (Miyata et al., 2007). This observation is supported by a study confirming that PNNs inhibit the internalization of exogenous tau protein in organotypic slice cultures of native mice (Suttkus et al., 2016). Another study demonstrated that PNNs are largely unaffected in a transgenic mouse model of Alzheimer disease (Morawski et al., 2010). Manipulating PNNs in order to enhance the synaptic plasticity and ameliorate possible cognitive dysfunctions might increase the sensitivity of GABAergic interneurons to neurotoxicity. Therefore, the point in time when the manipulation of perineuronal nets is attempted could be critical.

Recent studies revealed interesting new aspects about PNNs in Alzheimer disease. First, Crapser and colleagues demonstrated that microglia facilitate the loss of perineuronal nets in a transgenic mouse model for Alzheimer disease and in patients (Crapser et al., 2020). Therefore, manipulating microglia might consequently exert effects on the integrity and stability of PNNs (Figure 4). An additional study addressed this aspect utilizing a light entrainment approach. Here, it was shown that a light flickering at 60 Hz induced a remodeling of perineuronal nets in the visual cortex *via* microglia recruitment (Venturino et al., 2021). The study demonstrated that the activity of neurons was decisive for the microglia behavior and had an impact on them *via* purinergic receptors (Venturino et al., 2021). Based on these observations a modification of PNNs in Alzheimer patients might be achieved *via* a non-invasive procedure. A similar technique has already been used in an Alzheimer disease mouse model and revealed that a 40 Hz light-flickering reduced Aβ-levels in the visual cortex of pre-depositing mice (Iaccarino et al., 2016). A second study revealed that PNNs of Alzheimer disease patients undergo a modulation concerning the sulfation pattern of PNN-associated chondroitin sulfate glycosaminoglycans (CS-GAGs; Logsdon et al., 2022). Here, the authors could show that with ongoing disease progression (Braak staging of Alzheimer disease) the amount of non-sulfated glycosaminoglycans of the CS-O type is decreasing while the amount of sulfated CS-C and CS-E glycosaminoglycans is accumulating (Logsdon et al., 2022). In conclusion, the PNNs of Alzheimer patients undergo a progressing hypersulfation. In rats it has been shown that with an ongoing age the amount of the CS-C glycosaminoglycans is decreasing and replaced by CS-A, which renders the PNNs restrictive for axon growth (Foscarin et al., 2017). Furthermore, it is known that the double-sulfated CS-E strongly binds sema3a, a chemorepulsive guidance molecule (Dick et al., 2013). The targeting of PNN-associated CS-GAGs in Alzheimer patients might offer a novel interesting approach, as indicated by the study of Yang and colleagues, which used antibodies to 4S-GAGs to improve memory (Yang et al., 2017). Another interesting point is that the altered sulfation pattern of CS-GAGs might influence the binding properties of *Wisteria floribunda agglutinin* (WFA) which is frequently used for the visualization of perineuronal nets. Therefore, the reduced labeling of PNNs in *postmortem* brains of Alzheimer patients might not necessarily indicate a loss of perineuronal nets but rather an altered labeling pattern, as extensively discussed by Scarlett et al. (2022).

Consequently, PNN loss may serve as an endogenous compensatory mechanism to induce a higher degree of synaptic plasticity, but it may also interfere with neuroprotection. The manipulation of PNNs and ECM for treating cognitive impairments in Alzheimer’s disease is promising, but requires further study to avoid compromising other vital PNN functions.

### 4.3. Role of PNN-carrying interneurons in the demyelinating disease multiple sclerosis

Multiple sclerosis (MS) is the most common autoimmune disease in the world and more than 2.8 million people are affected, with increasing incidence (Sospedra, 2018; Walton et al., 2020). During the course of the disease pro-inflammatory T-lymphocytes are activated and expand in the CNS, where B-cells act as antigen presenting cells and pro-inflammatory cytokines are released by microglia and macrophages (Morandi et al., 2017; Jelcic et al., 2018; van Langelaar et al., 2020). In the following the myelin sheaths, which surround the axons of the neurons are the targets of these immune cells. In general, myelin sheaths are extensions of oligodendrocytes and are known to be important for the signal transmission and energy metabolism of axons (Hirrlinger and Nave, 2014; Poitelon et al., 2020). Acute lesions in the CNS are generally characterized by the demyelination of axons, inflammation and gliosis, which in the end leads to axonal degeneration and death (Franklin and Ffrench-Constant, 2008; Hagemeier et al., 2012; Lee et al., 2012; Motavaf et al., 2017). Since the spontaneous remyelination is insufficient, demyelination can occur so that the neurons ultimately die and acute lesions arise. A detailed description of MS and remyelination mechanisms has been provided elsewhere (Thompson et al., 2018; Lubetzki et al., 2020).

Due to the fact that MS can manifest in three different variants, namely relapsing remitting MS (RRMS), secondary progressive MS (SPMS) and primary progressive MS (PPMS), there are many different treatment options which can be individually tailored to the needs of the patient (Weiner, 2008; Ghasemi et al., 2017; McGinley et al., 2021). In cases of SPMS, a reduced concentration of GABA is associated with the degree of physical disability of the patients (Cawley et al., 2015). Here, multiple linear regression models were applied to investigate the relationship between cognitive functions and physical disability with reference to the GABA-level concentration ranges, justified for age, gender and N-acetyl-aspartate and glutamine-glutamate complex levels. The results ascertained that MS patients possessed significantly lower GABA-concentrations in the brain (Cawley et al., 2015). Especially in the hippocampus and in the sensorimotor cortex GABA-levels were significantly reduced in patients with MS. Furthermore, their motor and sensory performance were impaired in comparison with healthy subjects, as it was also confirmed in further independent studies (Cawley et al., 2015; Cao et al., 2018; Yang et al., 2022). Therefore, GABA-levels can be used as a specific biomarker for neurodegeneration, among other established techniques like proton magnetic resonance spectroscopy (Bai et al., 2015; De Stefano and Giorgio, 2015; Riese et al., 2015; Nantes et al., 2017; Gao et al., 2018). When focusing on GABA-receptor subtypes, it became clear that both GABA_A_- and GABA_B_-receptors play different roles in patients with MS. Concerning this aspect, it could be shown that the treatment with alemtuzumab, a well-established CD52 monoclonal antibody that significantly reduces the symptoms in RRMS patients, leads to a GABA_A_-receptor autoimmunity (Devonshire et al., 2018; Maniscalco et al., 2020). However, it could be also shown that axonal cues are driven by a GABA_A_-receptor regulation in oligodendrocytes and thereby play a crucial role in myelination and axon-glia recognition (Arellano et al., 2016). GABA_B_-receptors, on the other hand, are evenly expressed by oligodendrocyte precursor cells *in vivo* and *in vitro*, and the mediated signaling promotes finally OPC differentiation and myelin formation. Consequently, remyelination can also be promoted by the activation of GABA_B_-receptors (Serrano-Regal et al., 2020). Taken together, it appears that GABAergic signaling can be very useful as a therapeutical key regulator in myelin plasticity and in myelin repair, mainly because the GABA-receptors are expressed by myelinating cells (Serrano-Regal et al., 2020; Reyes-Haro et al., 2021).

In order to accelerate the signal conduction, the myelin in the CNS is segmented into internodes and nodes of Ranvier (Harris and Attwell, 2012; Arancibia-Carcamo and Attwell, 2014). In addition to the segmentation of myelin into internodes and nodes of Ranvier, the axons of neurons are also covered by ECM molecules that form axonal coats. ECM axonal coats play an important role in the signal transmission and energy metabolism of axons, and alterations of the ECM composition along the axon has been implicated in various neurological disorders, including multiple sclerosis (van Horssen et al., 2007; Bruckner et al., 2008; Morawski et al., 2012; Lendvai et al., 2013). In addition, the ECM plays an important role in myelination and remyelination. Hyaluronan and CSPGs as an example are upregulated at the MS lesion site (Back et al., 2005; Keough et al., 2016). Through immunolabeling in the optic nerve of mice, it could be shown that certain ECM molecules like Brevican, Neurocan, Versican, and TnR as well as integral PNNs are attached to the nodes of Ranvier through neurofascin (NF186), a cell adhesion molecule (Susuki and Rasband, 2008; Oohashi et al., 2015; Fawcett et al., 2019). Interestingly, a commonality of PNNs and myelin sheaths has been observed in the past, in that PNNs have been shown to ensheath minor populations of excitatory neurons in mice, rat and in humans (Mauney et al., 2013; Lensjo et al., 2017; Ueno et al., 2018; Bucher et al., 2021). By analyzing rodent and human tissue it could be shown that two subtypes of GABAergic interneurons are myelinated, namely fast-spiking parvalbumin-expressing interneurons and somatostatin-expressing interneurons (Mazuir et al., 2021). Thereby, these inhibiting myelinated interneurons can be detected in the cortex and hippocampus (Jinno et al., 2007; Micheva et al., 2016, 2018). Parvalbumin is mainly expressed by two main types of interneurons, namely basket cells and chandelier cells, that are interconnected by gap junctions and synapses in laminar networks (Bucher et al., 2021). The maturation of the parvalbumin network is functionally and physically stabilized by axonal myelination and PNNs (Fawcett et al., 2019; Bucher et al., 2021). Recently, it was investigated whether parvalbumin could be used as a specific marker of grey matter neurodegeneration in MS patients by analyzing the cerebrospinal fluid. Here, it was found out that especially inhibitory interneurons are damaged in people with MS (Magliozzi et al., 2021). This result was surprising since it was believed for a long time that interneurons are usually not surrounded by myelin sheaths or only by sparse ones with minor functional significance. Nowadays, it is known that GABAergic interneurons in the cerebral cortex of mice and humans are clearly myelinated. This is particularly the case for the axons of parvalbumin-expressing neurons (Stedehouder et al., 2017; Stedehouder and Kushner, 2017). However, the exact function of these myelin sheaths is not understood, neither why interneurons are insulated by myelin layers (Fawcett et al., 2019; Dubey et al., 2022).

Taken together, it became clear that in the future especially the individual ECM components of PNNs might be utilized as helpful modifiers of neuroinflammation and myelination processes in multiple sclerosis (Ghorbani and Yong, 2021; Browne et al., 2022). So far, several proteins of the PNNs were shown to have an influence on myelination processes in general, so that integral PNNs could serve as possible therapeutic targets for future MS research.

### 4.4. Role of PNN-carrying interneurons in epilepsy

Epilepsy is one of the most well-known disorders of the CNS that goes along with an E/I-imbalance caused by an increased excitation, a decreased inhibition or by a combination of both (Fritschy, 2008). However, this traditional point of view has recently been questioned because genetic mutations were identified that are associated with epilepsy, but do not directly influence the E/I-balance. Furthermore, a treatment of epileptic patients with medications that either decrease excitation or increase inhibition does not necessarily show beneficial effects, especially during development (Shao et al., 2019; Righes Marafiga et al., 2021). Notwithstanding, GABAergic interneurons play an important role for this pathological condition and are affected in patients with epileptic seizures (Arellano et al., 2004). Studies utilizing *postmortem* human tissue revealed disruptions of parvalbumin-expressing interneurons in the cortex and in the hippocampus (Sloviter et al., 1991; Ferrer et al., 1994; Arellano et al., 2004; Andrioli et al., 2007). While a strong reduction of parvalbumin-expressing interneurons could be shown in this context, the perisomatic inhibitory input of dentate granule neurons was unchanged in temporal lobe epilepsy (Wittner et al., 2001). Therefore, it was hypothesized that the loss of the parvalbumin immunoreactivity possibly indicates rather a disruption of the parvalbumin synthesis than an interneuron loss (Zaitsev, 2016).

Based on findings that PNNs regulate the synaptic balance of structural excitatory and inhibitory synapses on parvalbumin-expressing interneurons these dense ECM-structures are interesting for epilepsy research (Gottschling et al., 2019; Wegrzyn et al., 2021a; Mueller-Buehl et al., 2022). Indeed, several review articles have summarized and discussed possible implications of PNNs for the pathophysiology of epilepsy (McRae and Porter, 2012; Wen et al., 2018; Chaunsali et al., 2021). A recently published study generated a conditional knockout of *Depdc5*, a gene highly involved in the target of rapamycin (mTOR) pathway and furthermore one of the most causative genes in patients with epilepsy and malformations of the cortex (Yang et al., 2023). A loss of PNNs was observed, which was accompanied by a reduction in parvalbumin-expressing interneurons (Yang et al., 2023). Importantly, the authors could verify a microglia-induced proteolytic degradation of PNNs before the seizures occurred. Another recently published study could show that PNNs specifically decrease the membrane capacitance of fast-spiking parvalbumin-expressing interneurons acting thereby as electrostatic insulators (Tewari et al., 2018). This characteristic of PNNs enables parvalbumin-expressing interneurons to fire potentials at high frequencies. The authors could observe that a degradation of PNNs increased the membrane capacitance and consequently reduced the firing of GABAergic interneurons, facilitating the hyperactivity of excitatory neurons (Tewari et al., 2018). Based on their findings, Tewari and colleagues assume that the disruption of PNNs possibly drives the E/I-imbalance in epilepsy. This assumption is supported by a study that subjected brain slices to ChABC treatment and detected a reduced excitability of cortical parvalbumin-expressing interneurons (Balmer, 2016). As previously described in the context of Alzheimer disease, the sulfation pattern of PNNs can modify their properties (Mikami and Kitagawa, 2023). Concerning this aspect, a previously published study verified an increase of chondroitin 6-sulfation and chondroitin 6-sulfation-enriched PNNs in the murine cortex and hippocampus after kainic acid treatment (Yutsudo and Kitagawa, 2015).

The question of how PNNs are disrupted in epilepsy was addressed in previous studies that unraveled an involvement of matrix metalloproteinases (MMPs). In a chemo-convulsant rodent epilepsy model it has been shown that the *status epilepticus* induced a proteolysis of aggrecan by MMPs that was accompanied by a fragment accumulation in the hippocampus (Rankin-Gee et al., 2015). Furthermore, the MMP-mediated cleavage of aggrecan was concentrated especially around parvalbumin-expressing interneurons and went along with a reduced expression of PNN-components like HAPLN1 and hyaluronan synthase 3 (HAS3; McRae et al., 2012). In line with these observations, the inhibition of matrix metalloproteinases prevented the generation of epileptic seizures in a kindling model for epilepsy (Pollock et al., 2014). In conclusion, an increasing number of studies unraveled interesting new aspects concerning the function of PNNs and their possible role for epileptic seizures. Based on these observations a better comprehension of this disorder has been obtained, which possibly allows for novel therapeutical strategies in future.

## 5. Conclusion

Parvalbumin-expressing interneurons play a crucial role concerning the maturation of inhibitory GABAergic circuits, which are essential for optimal adaptation to incoming sensory information during critical periods. The condensation of the ECM of the matrisome as PNNs around interneurons acts as a molecular brake for the critical period, inhibiting axonal sprouting and binding recognition molecules to decrease plasticity. PNNs have also been identified as important regulators of synaptic stability and E/I-balance. Accordingly, disruptions of PNNs in neurodegenerative and psychiatric diseases may affect the synaptic balance on parvalbumin-expressing interneurons and their electrophysiological properties. In this context, manipulating PNNs and ECM components may serve as a promising therapeutic target for treating cognitive impairments and neuroinflammation and may lead to novel therapeutic strategies in the future. However, it should be noted that much of the information on PNN functions stems from studies in which PNNs were enzymatically digested, or from knockout mutants lacking individual PNN components. However, the majority of PNN components also occur in a soluble form in the extracellular space. Therefore, the effects of PNN degradation or knockout cannot strictly be attributed solely to modifications of PNN-resident components. The resulting changes of the interstitial and perisynaptic ECM are rarely addressed in the literature. Consequently, an important challenge in the future will be to find a way to manipulate exclusively PNNs, without affecting neighboring compartments of the ECM.

## Author contributions

CM-B, DW, and AF contributed to the conceptualization of the manuscript. CM-B, DW, JB, and AF drafted the original manuscript. CM-B and AF revised and edited the manuscript. CM-B performed the experiments and created the figures. AF organized the funding acquisition. All authors contributed to the article and approved the submitted version.

## Funding

The work was funded by the German Research Foundation (DFG FA 159/22-1N° 290189690 and DFG FA 159/24-1N° 407698736 to AF). We acknowledge support from the DFG Open Access Publication Funds of the Ruhr-Universität Bochum.

## Conflict of interest

The authors declare that the research was conducted in the absence of any commercial or financial relationships that could be construed as a potential conflict of interest.

## Publisher’s note

All claims expressed in this article are solely those of the authors and do not necessarily represent those of their affiliated organizations, or those of the publisher, the editors and the reviewers. Any product that may be evaluated in this article, or claim that may be made by its manufacturer, is not guaranteed or endorsed by the publisher.

## Figures and Tables

**Table 1:** 

5HT3_a_R	5-Hydroxytryptamine 3a receptor
AMPA	α-Amino-3-hydroxy-5-methyl-4-isoxazolepropionic acid
Aβ	β-Amyloid
Arx	Aristaless related homeobox
APP	Amyloid precursor protein
CNS	Central nervous system
ChABC	Chondroitinase-ABC
CS-A	Chondroitin-4-sulfate
CS-C	Chondroitin-6-sulfate
CS-E	Chondroitin-4-6-sulfate
CS-GAGs	Chondroitin sulfate glycosaminoglycans
Dlx1/2	Distal-less homeobox-1/2
BM	Basement membrane
ECM	Extracellular matrix
E/I	Excitatory/inhibitory
GABA	γ-Aminobutyric acid
Gsx1	Gs Homeobox 1
Gsx2	Gs Homeobox 2
HAPLN	Hyaluronan and proteoglycan binding link protein family
HAS	Hyaluronan synthase
Lhx6	Lim Homeobox 6
Lhx8	Lim Homeobox 8
LTP	Long term potentials
Mash-1	Mammalian achaete-scute homolog-1
MMP	Matrix metalloproteinase
MS	Multiple sclerosis
Nkx2.1	Nk2 homeobox 1
NPY	Neuropeptide Y
Nkx5.1	Nk5 homeobox 1
NMDAR	N-methyl-D-aspartate receptor
OTX2	Orthodenticle homeobox protein 2
Oxr1	Oxidative resistance 1
PNN	Perineuronal net
PPMS	Primary progressive multiple sclerosis
RRMS	Relapsing remitting multiple sclerosis
SPMS	Secondary progressive multiple sclerosis
Sema3A	Semaphorin 3A
Shh	Sonic hedgehog
TNC	Tenascin-C
TNR	Tenascin-R
WFA	*Wisteria floribunda* agglutinin
